# AFLP-based genetic mapping of the “bud-flowering” trait in heather (*Calluna vulgaris*)

**DOI:** 10.1186/1471-2156-14-64

**Published:** 2013-08-02

**Authors:** Anne Behrend, Thomas Borchert, Monika Spiller, Annette Hohe

**Affiliations:** 1 Department Plant Propagation, Leibniz-Institute of Vegetable and Ornamental Crops (IGZ), Kuehnhaueser Strasse 101, 99090, Erfurt, Germany; 2 Present address: Siemens Healthcare Diagnostics Holding GmbH, Ludwig-Erhard-Straße 12, 65760, Eschborn, Germany; 3 Department Molecular Plant Breeding, Leibniz University Hannover, Herrenhaeuser Strasse 2, 30419, Hannover, Germany

**Keywords:** Bud-bloomer, Flower architecture, Linkage map, ML mapping, Molecular marker

## Abstract

**Background:**

*Calluna vulgaris* is one of the most important landscaping plants produced in Germany. Its enormous economic success is due to the prolonged flower attractiveness of mutants in flower morphology, the so-called bud-bloomers. In this study, we present the first genetic linkage map of *C. vulgaris* in which we mapped a locus of the economically highly desired trait “flower type”.

**Results:**

The map was constructed in JoinMap 4.1. using 535 AFLP markers from a single mapping population. A large fraction (40%) of markers showed distorted segregation. To test the effect of segregation distortion on linkage estimation, these markers were sorted regarding their segregation ratio and added in groups to the data set. The plausibility of group formation was evaluated by comparison of the “two-way pseudo-testcross” and the “integrated” mapping approach. Furthermore, regression mapping was compared to the multipoint-likelihood algorithm. The majority of maps constructed by different combinations of these methods consisted of eight linkage groups corresponding to the chromosome number of *C. vulgaris*.

**Conclusions:**

All maps confirmed the independent inheritance of the most important horticultural traits “flower type”, “flower colour”, and “leaf colour”. An AFLP marker for the most important breeding target “flower type” was identified. The presented genetic map of *C. vulgaris* can now serve as a basis for further molecular marker selection and map-based cloning of the candidate gene encoding the unique flower architecture of *C. vulgaris* bud-bloomers.

## Background

**Figure 1:**
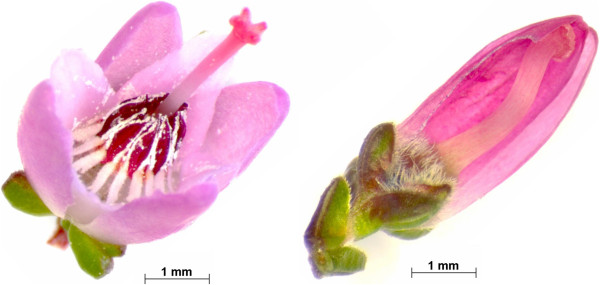
***C. vulgaris *flower types.** Flowers from a segregating population developed at the IGZ, A – wild-type flower, flower organs from centre to outer edge: carpels, stamens, petals, sepals, bracts, B - bud-bloomer, closed perianth partly removed, flower organs from centre to outer edge: carpels, sepals, sepals, bracts.

*Calluna vulgaris* is a woody landscaping plant from the Ericales order with rising economic importance in Northern Europe. Here, Germany is a major producing and exporting country. In 2010, 110 million plants of *C*. *vulgaris* have been produced in Germany [1]. Its high popularity is due to the introduction of mutants with extended flowering time into commercial breeding. The so-called “bud-bloomers” (or “bud-flowering” phenotypes [2]) were probably derived from two British clones collected in 1936 and 1948 and several individuals found in natural populations in 1970 in the Netherlands [1]. Today, bud-bloomers make up the major market share of *C*. *vulgaris* compared to cultivars with wild-type and filled flowers because of their delayed flower senescence. More than 80% of all varieties that are protected in Germany are of the bud-flowering phenotype [3]. This phenotype is composed of three distinctive traits [4] and so far it has not been documented in any other plant species: the perianth remains closed during anthesis, petals are transformed into petaloid sepals, and stamens are completely missing (Figure 1). Since the perianth organs shield the unfertilised stigmas from cross-pollination and self-pollination is impossible due to the lack of stamens, pollination is impeded and the flowers do not show senescence during the flowering season until winter.

Despite their commercial importance, the inheritance of the most important breeding targets “flower type”, “flower colour”, and “leaf colour” has rarely been studied or been systematically used so far during the relatively short breeding history of *C*. *vulgaris*. The bud-flowering phenotype in *C*. *vulgaris* has been identified as a mono-genic recessive trait; [5] however, until now its genetic basis is unclear.

Our aim was to develop a genetic map of *C*. *vulgaris* which is on the one hand a useful tool to locate horticultural traits of interest and elucidate their inheritance. On the other hand, it will serve as a framework for candidate gene cloning to identify the genetic basis of the bud-flowering trait in *C*. *vulgaris*. Although RAPD (Random Amplification of Polymorphic DNA) and ISSR (Inter Simple Sequence Repeat) fingerprinting has been used before to analyse the genetic diversity among *C*. *vulgaris* genotypes and to generate a reliable system to identify Essential Derived Varieties in *C*. *vulgaris*[6], this is the first mapping approach in this species. *C*. *vulgaris* is a heterozygous cross-pollinating plant. It is a diploid species with a chromosome set of 2n = 2× = 16 [7-9]. The DNA-content was determined to be 1.18 pg/2C [4]. Data on the DNA-sequence of *C*. *vulgaris* is very limited and the AFLP (Amplified Fragment-Length Polymorphism) procedure has already been adapted for *C*. *vulgaris*[10]. Hence, AFLPs have been chosen for the rapid generation of markers covering the genome. The only plausible way to evaluate the genetic map of a non-sequenced organism like *C*. *vulgaris* is to compare different maps [11]. Since only a single mapping population was considered, different mapping approaches and algorithms were used.

## Results

### AFLP markers and segregation patterns

In a previous study, the discriminative power and resolution of AFLPs in *C*. *vulgaris* was found to be optimal with double-digestion by HindIII and MseI, a preamplification using non-selective and non-labelled primers and selective amplification with three selective bases at the 3′-end of the HindIII and MseI primers [10]. HindIII and MseI primer (+3/+3) or (+2/+3) combinations resulting in most polymorphic bands were chosen from the pre-test for the mapping approach and their reproducibility was proven. The methylation-sensitive enzyme HhaI was added to the protocol to prevent clustering of AFLP markers in telomeric or centromeric regions and enrich non-methylated single copy, gene-rich regions [12]. On average, each primer combination resulted in nine polymorphic markers in the mapping population. HhaI/HindIII primer combinations yielded 8.36 polymorphic markers per primer combination. This number was slightly lower than 9.88 polymorphic markers per primer in MseI/HindIII primer combinations.

Each of the primer combinations Hhal-CA-HindIII-CAT/Hhal-CAA-HindIII-CAT, Hhal-CA-HindIII-AGT/Hhal-CAA-HindIII-AGT, Hhal-CA-HindIII-CGA/Hhal-CAA-HindIII-CGA,  MseI-TCG-HindIII-CA/MseI -TCG-HindIII-CAT,  MseI-TCG-HindIII- AC/MseI-TCG- HindIII-ACA,  MseI-TCG-HindIII- CA/MseI-TCG-HindIII-CAT, MseI-CAC-HindIII-AC/MseI-CAC-HindIII-ACA, and MseI-CAC-HindIII-CA/MseI-CAC-HindIII-CAT resulted in two different markers with identical segregation patterns because the primer combinations amplified the same sequence. Overall, 29% of all amplified markers of these primer +2-primer+3 combinations were found to show an identical segregation pattern compared to markers obtained by the corresponding primer+3-primer+3 pair. The primer+2-primer+3/primer+2-primer+3 combinations  HhaI-AA-HindIII-AAC/HhaI- CA- HindIII- AAC, HhaI-AC-HindIII-CGA/HhaI-CC-HindIII-CGA, HhaI-CC-HindIII-CAT/HhaI-AC-HindIII-CAT,  HhaI-CA-HindIII-ACT/HhaI-AA-HindIII-ACT also amplified identical loci. Here, 21% of the markers generated with the first primer+2/primer+3 combination listed above showed an identical segregation pattern and similar fragment sizes as markers generated with the second +2/+3 primer pair. This is probably due to mismatches at the 3′-end of the primers since *Taq* polymerase lacks a 3′ to 5′ proofreading activity.

The marker scoring was performed completely for all genotypes. Therefore, the data set did not contain missing values. In total, 659 polymorphic markers have been identified. From these, 84 markers were excluded because they were neither found in the male (‘F1’) nor in the female crossing partner (‘Maria’) but were segregating in the mapping population. Additionally, 40 markers with redundant segregation patterns were removed. The 535 remaining markers were coded according to their origin as <lmxll> (39.1%) for maternal (heterozygous in the female crossing partner), as <nnxnp> (34.4%) for paternal (heterozygous in the male crossing partner), and as <hkxhk> (26.5%) for biparental markers (heterozygous in both crossing partners). Segregation distortion was observed for maternal, paternal and biparental markers (Table 1). Overall, 330 markers were considered as undistorted (Table 1, **bold** type). From these, the expected segregation ratio of 1:1 was met by 67.5% of the maternal markers and 67.4% of the paternal markers, whereas only 45.8% of the biparental markers matched the expected segregation ratio of 3:1 (Table 1).

**Table 1:** Indication of markers

**Marker category**	**Markers scored**	**Segregation 1:1 [%]**	**Segregation 3:1 [%]**	**Odd segregation [%]**
<lmxll>	209	**140 [67.0%]**	45 [21.5%]	24 [11.5%]
<nnxnp>	184	**124 [67.4%]**	18 [9.8%]	42 [22.8%]
<hkxhk>	142	43 [30.3%]	**66 [46.5%]**	33 [23.2%]
Sum	535	307	129	99

Number and segregation ratio of markers categorised as maternal (<lmxll>), paternal (<nnxnp>) and biparental (<hkxhk>); percentages are calculated separately for the three segregation types; markers showing undistorted segregation are marked in **bold** type.

All phenotypic markers passed the *χ*^2^-test for 1:1 segregation in the mapping population. Green leaf colour was found in 63 individuals, yellow foliage in 61 plants. 58 plants displayed the bud-flowering phenotype and 66 showed wild-type morphology. Phenotyping of the flower colour defined 60 individuals as white-flowering and 64 as pink. Plants with pink flower also had blushed shoot tips. Obviously, the three traits “flower type”, “flower colour”, and “leaf colour”, were not linked to each other.

### Estimation of linkage groups

The mapping population resulted from combining the products of independent meiosis in both parents. Therefore, the data set contained segregating markers inherited by the female parent, segregating markers inherited by the male parent and segregating markers inherited by both parents. Two ways of linkage group estimation were tested. In the pseudo-testcross (PTC) approach, the data set was split in two subsets: maternal with biparental markers and paternal with biparental markers. A separate map was constructed from each subset, resulting in one map of the female crossing partner and one of the male crossing partner. Using the biparental markers in both parental maps as anchor points, corresponding linkage groups from the parental maps were manually integrated. In the mapping software JoinMap 4.1, the PTC approach can only be combined with the regression mapping algorithm (RG). In contrast, the “integrated” approach was combined with RG mapping and the multipoint maximum likelihood (ML) mapping algorithm.

**Figure 2:**
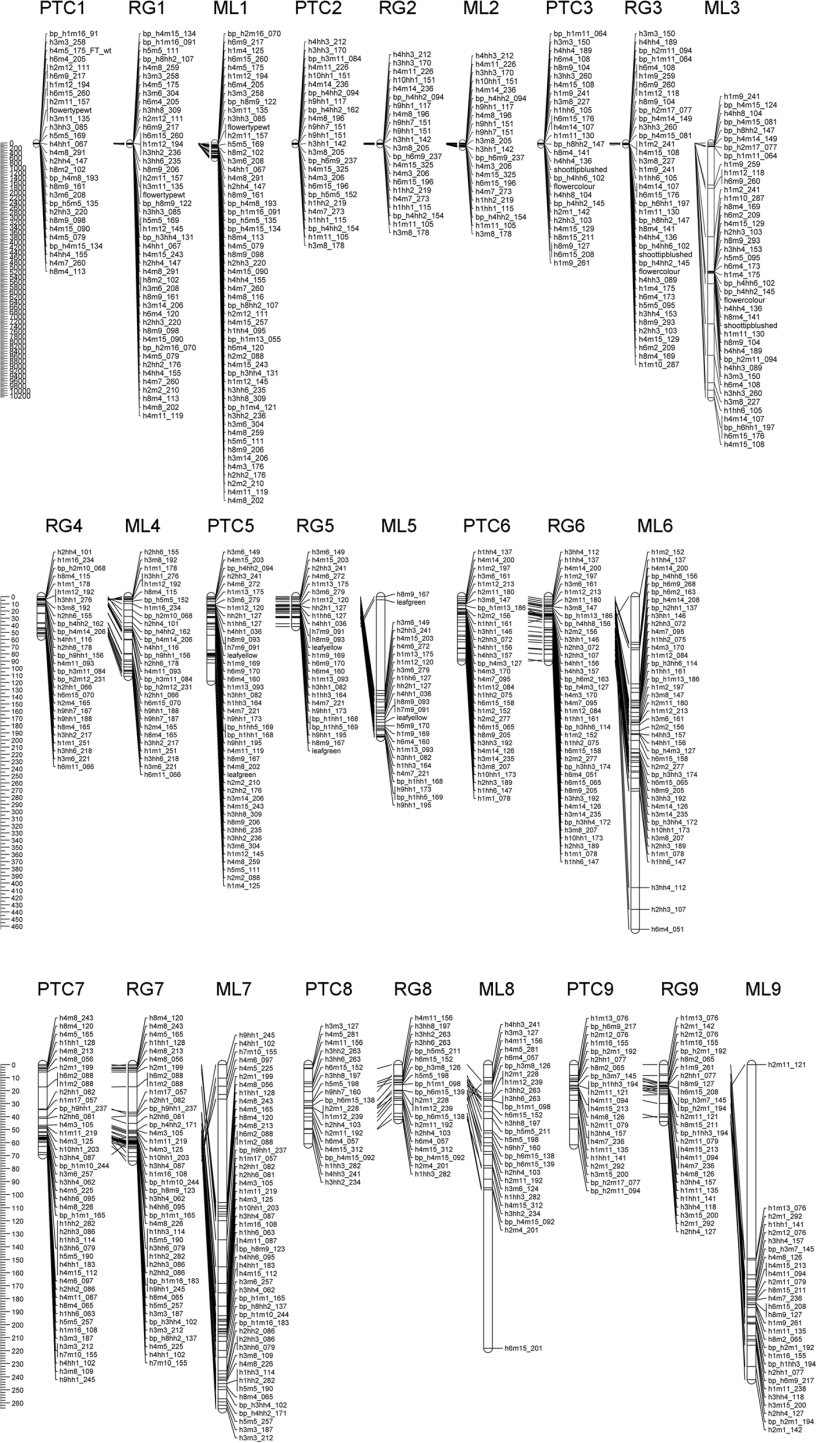
**Collinearity of maps.** Alignment of linkage groups (LG) from the PTC and the “integrated” approach followed either by RG or by ML mapping, lines between linkage groups indicate homologous loci, presented maps were drawn using MapChart 2.2 [13].

Due to the published chromosome number (2n = 2× = 16), eight linkage groups were expected for *C*. *vulgaris*. Based on all markers showing the expected segregation ratio (data set 1), nine linkage groups were constructed in the “integrated” approach, but eight linkage groups were derived with the PTC approach in which linkage group 4 was missing (Figure 2, Table 2). The addition of the first group of distorted markers (data set 2, Additional files 1, 2, and 3) resulted in eight linkage groups in both mapping strategies since in the integrated approach, linkage group 9 did not form. After the addition of all distorted markers (data set 3, Additional files 1, 2, and 3), the number of linkage groups obtained by the “integrated” approach was stable, but linkage group 9 was lost (in addition to the missing linkage group 4) in the PTC approach, resulting in only seven linkage groups using this mapping strategy.

**Table 2:** Comparison of linkage groups

	**Integrated RG**		**PTC RG**		**Integrated ML**	
Linkage group	Length in cM	Number of loci	Length in cM	Number of loci	Length in cM	Number of loci
1	68.2	46	49.5	29	616	56
2	70	22	68.2	25	173.7	22
3	82.1	28	83.2	28	10283.8	42
4	55.1	28			111.4	29
5	41.9	26	118.1	43	196.1	26
6	90.6	40	89.8	32	464.3	40
7	74.2	43	69.3	45	264.5	50
8	42.2	20	61	20	218.2	27
9	43.8	27	61.7	22	242.1	29
Total	581.2	252	601.1	243	12570.1	321
Genome coverage	224.1%		83.2%		227.7%	
cM/Mb Ratio	5		5.2		109.3	

Main characteristics of the linkage groups (size, loci number, calculated genome coverage) in the genetic maps from “integrated” RG mapping, PTC and “integrated” ML mapping; map length in cM.

In the “integrated” approach, nearly all of the undistorted markers in data set 1 were mapped. In contrast, the PTC mapping approach left a substantial fraction of markers unassigned to linkage groups (Table 2). In both mapping approaches, biparental markers tended to be eliminated from the linkage groups at higher LOD scores, which was disadvantageous for map integration in the PTC approach because biparental markers serve as anchor markers. Although some exceptions were observed, in most cases the addition of distorted markers prolonged the map length of individual linkage groups and increased the number of mapped loci irrespective of the mapping strategy (Additional files 1, 2, and 3). After addition of distorted markers, grouping was not substantially changed, thus segregation distortion did not show a major impact on the grouping. Distorted markers neither formed a distinct linkage group, nor did they accumulate in certain areas of the existing linkage groups. Hence, chromosomal or meiotic drive is no plausible explanation for the localisation of distorted markers on the map.

### Comparison of mapping strategies

The map characteristics resulting from different mapping strategies using the data set containing only markers with undistorted segregation (data set 1) are summarised in Table 2. Based on the “integrated” RG approach, nine linkage groups with an average length of 61.2 cM, an average marker number of 28 per linkage group, an average genetic distance of 5 cM per 1 Mb, and twofold genome coverage were initially constructed. The lowest number of markers was mapped using the PTC approach. The average length of linkage groups from the PTC approach was 75.1 cM; the average number of markers per linkage group was 30.4, and the genetic distance was 5.2 cM per 1 Mb. Full genome coverage was not reached using the PTC approach. 321 markers were mapped by the “integrated” approach followed by ML mapping. The average map length per linkage group using the “integrated” ML strategy was increased to 1,396.7 cM; each linkage group contained 36 markers on average. The drastic increase of map length was caused by the inflated length of linkage group 3 carrying the phenotypical markers for flower colour and colour of shoot tip (Figure 2, Table 2). This is probably provoked in the ML mapping algorithm by large gaps between uniparental and biparental markers positioned at the end of the linkage group. The marker density in ML mapping was low (one marker every 39.1 cM) compared to the other mapping approaches (RG: one marker per 2.3 cM, PTC: one marker per 2.5 cM). Additionally, a twofold genome coverage was calculated in this approach. Maps from the “integrated” approach combined with the ML algorithm by far showed the longest map distances. The maximum distance between two loci was 19.6 cM using “integrated” and 37.5 cM using the PTC approach. In contrast, the largest gap between two loci on the ML map was 1,954 cM leading to the genetic distance of 109.3 cM per 1 Mb.

The addition of distorted markers slightly increased the map length in the “integrated” RG as well as in the PTC approach (Additional files 1 and 2). Regarding data set 1, the map calculated by the PTC approach contained fewer markers compared to “integrated” RG mapping. After the addition of distorted markers, the number of integrated distorted markers on the PTC maps was higher than on “integrated” RG maps. In ML mapping, the map length was quadrupled by mapping all markers segregating 1:1 and 3:1. By addition of all odd markers, the map length was even extended to 64,300 cM. Hence, the ratio of genetic map distances to physical map distances (cM/Mb ratio) of ML maps was drastically higher than the values calculated for the “integrated” RG map and the PTC map (Additional files 1, 2, and 3). The cM/Mb ratio of ML maps extremely increased with the addition of distorted markers since in this mapping approach all available markers were assigned to linkage groups (Additional file 3). Therefore, the extreme length of ML maps after addition of odd markers points out the poor fitting of these markers. The mapping approaches “integrated” and PTC were compared in combination with the RG mapping algorithm (data set 1). The calculation of the map length ratios resulted in overall slightly higher values for PTC linkage groups (Table 3). Since in the PTC approach eight linkage groups were calculated, linkage group 4 was left unmatched between the PTC and the “integrated” approach. In the PTC approach, maternal markers, which were assigned to linkage group 4 in the “integrated” approach, were insufficiently linked to biparental markers; thus, map integration was not possible. The comparison of loci mapped in all other linkage groups resulted in a congruency between 61% (linkage group 1) and 86% (linkage group 6). The order of loci on the map appeared to be well-preserved in all linkage groups (Figure 2).

**Table 3:** Comparison of the mapping approaches

**Linkage group**	**Length ratio PTC/”integrated”**	**Common loci PTC/”integrated”**
1	0.73	61%
2	0.97	89%
3	1.01	72%
4		0
5	2.82	70%
6	0.99	86%
7	0.93	73%
8	1.46	72%
9	1.41	78%
Total	1.09	73%

Both “integrated” and PTC mapping approaches were combined with the RG mapping algorithm: ratio of map lengths and proportion of loci from the “integrated” RG map, which are also mapped in the corresponding linkage group of the map calculated with the PTC approach (data set 1).

Comparing the ML and RG mapping algorithms in combination with the “integrated” mapping approach (data set 1), the numbers of common markers varied between 82% and 100% (Table 4). Maps constructed using the ML mapping strategy were considerably longer than maps calculated by RG mapping and contained more loci. Linkage group 6 displayed the smallest difference in map length, being twofold longer in the map calculated by ML mapping compared to the RG algorithm. In contrast, the map lengths of linkage group 3 differed by a factor of 125. The order of markers on the different maps clearly differed in linkage groups 1 and 3, whereas it was almost identical in linkage groups 4, 5, 6, 7, and 8. For linkage group 2, an identical order of loci was observed. Major rearrangements of the marker order in the linkage groups were mainly observed in those linkage groups with a high number of biparental markers. However, even on linkage groups showing major rearrangements, the micro-order of closely linked loci was retained (Figure 2).

**Table 4:** Comparison of the mapping algorithms

**Linkage group**	**Length ration RG/ML**	**Common loci RG/ML**
1	9.03	82%
2	2.48	100%
3	125.26	98%
4	8.43	98%
5	2.66	95%
6	2.16	96%
7	3.26	92%
8	6.27	85%
9	5.05	96%
Total	22.15	92%

RG and ML mapping was combined with the “integrated” mapping approach: ratio of map lengths and proportion of loci from the “integrated” RG map, which are also mapped in the corresponding linkage group of the map calculated with the ML mapping algorithm (data set 1).

### Localisation of phenotypical markers

In all maps (Figure 2), the phenotypical markers for flower type (*flowertypewt*), flower colour (*flowercolor*), and leaf colour (*shoottipblushed*) each were located on different linkage groups. This is in correspondence with the observation of independent inheritance of these traits during scoring of phenotypical markers in the mapping population. The flower type was mapped in linkage group 1. The AFLP marker h2m11_157 was found to co-segregate with the highly desired character flower type in 124 genotypes. “Flower colour” and “shoot tip” colour were found on linkage group 3, whereas *leafgreen* and *leafyellow* clustered on linkage group 5. The genetic distance of the phenotypic markers *shoottipblushed* and *flowercolour* was 0.7 cM in the PTC approach and in the “integrated” approach combined with RG mapping, whereas it was 2.7 cM in the map calculated using ML mapping. In contrast, although expected, the alternative markers *leafgreen* and *leafyellow* for colour of foliage were not found at the same locus in maps derived from undistorted markers. With addition of distorted markers, the clustering of *shoottipblushed*/*flowercolour* and *leafgreen*/*leafyellow* improved in RG maps (data not shown). These two phenotypic marker pairs are considered as alternative alleles of single genes. Hence, *leafgreen* and *leafyellow* as well as *shoottipblushed* and *flowercolour* are supposed to be located at an identical locus each. After the addition of odd markers, the map distance of the loci *leafgreen*/*leafyellow* decreased in the PTC and RG maps. However, this improvement was not observed in ML mapping.

## Discussion

The main purpose of this work was to map the most important horticultural trait of *C*. *vulgaris*, the flower type, and to find possible molecular markers for this trait. Since almost no sequence information is available for *C*. *vulgaris* (as it is the case for most ornamental crops) and codominant SSR (Simple Sequence Repeats) or EST (Expressed Sequence Tag) markers have not been established yet, markers for genetic mapping were generated using the AFLP procedure. The number of polymorphic markers per primer pair was relatively low. In *Rhododendron simsii* hybrids, a genus also belonging to the Ericaceae, the number of polymorphic markers per primer combination was fivefold higher [14]. Hence, the reduced amount of polymorphism in *C*. *vulgaris* might be a consequence of a narrow gene pool with short genetic distances [6]. *C*. *vulgaris* has a comparatively short breeding history and crossbreeding with other species is impossible, since it is the only species in its genus. Thus, varieties are closely related to each other [6].

The mapping population was not specified as “BC1” (first generation backcross), because BC1-populations are defined as the result of backcrossing the F1 of a cross between two fully homozygous diploid parents to one of the parents [15]. Therefore, the cross-pollination (“CP”) type was chosen, which allows map construction from markers with different segregation ratios.

The phenotypcial markers segregated 1:1 in the mapping population, indicating a monogenetic recessive inheritance of the traits “flower type”, “flower colour”, “leaf colour” and “colour of shoot tip”. The genetic basis of the flower type is unclear, but homeotic genes controlling flower organ development according to the ABC model [16] are possible candidate genes, although no hypothesis exists, which of the floral organ identity genes affects flower opening in *C*. *vulgaris*. The trait “leaf colour” can be attributed to a chlorophyll deficiency in yellow-leafed plants. The pink flower colour is probably based on anthocyanin biosynthesis. The trait “colour of the shoot tip” was located on the same linkage group, because this character also depends on the anthocyanin production which is prominent in young, rapidly expanding tissues [17,18]. Accordingly, plants with pink flowers have blushed shoot tips. Since flower type, flower colour, and leaf colour were mapped in different linkage groups, these traits are inherited independently from each other and can be freely combined in breeding strategies.

Nearly 40% of the markers showed segregation distortion. AFLP markers are sensitive to segregation distortion, but even highly distorted markers have been used to construct genetic maps in other species [14,19,20], as marker order and map length were not severely affected [21]. Segregation distortion might be a consequence of natural phenomena like e.g. gametophytic self-incompatibility. Therefore, it is consequential to keep distorted markers in the data set and evaluate their localisation after map calculation. In these cases, distorted markers should cluster on separate linkage groups, a certain chromosome region [22,23], or should be attributed to one parental class [24]. However, in our maps, distorted markers were spread over all identified linkage groups. Likewise, the fraction of distorted markers was equal in the maternal and paternal data set. If odd segregation ratios were caused by gene conversion, equal numbers of under- and overrepresented markers (e.g. of maternal markers segregating 1:3 and 3:1) should be found [25] which is also not the case in our study. Here, markers segregating 3:1 were clearly overrepresented and different odd segregation ratios were obtained. Although a positive effect was observed by adding distorted markers on clustering of the alternative alleles *leafgreen*/*leafyellow* and *shoottipblushed*/*flowercolor* in RG maps, segregation distortion is regarded as a technical artefact in our study. Fragment complexes are known to lead to pseudo-distorted segregation ratios [26]. Equally sized fragments were amplified from different genome regions and co-migrated in the gel creating single bands containing several PCR products. Fragment complexes are the major concern of the AFLP technique and also termed homoplasy [27] or collision [28]. Hence, it cannot be excluded that fragment complexes behave like markers from a single region and by chance meet the expected 1:1 or 3:1 ratio. For example, a high ratio (41%) of distorted AFLP markers has been obtained in a data set from *Cryptomeria japonica* and was attributed to fragment complexes [29]. In the study on *Cryptomeria japonica*, segregation distortion was overcome by using four to five selective bases in AFLP primers for final amplification.

The construction of maps using the “integrated” approach was compared to the PTC approach in com-bination with the RG mapping algorithm. Both methods resulted in maps of comparable length; however, the number of loci per linkage group was lower in the PTC approach. Moreover, a major constraint of the PTC approach turned out to be the tendency to eliminate less informative biparental markers from the data set at high LOD scores, which was necessary in some cases to achieve stable groupings. This is especially critical in the PTC approach, as biparental markers serve as anchor markers for map integration. Moreover, segregation distortion could not be attributed to either parent. Therefore, the PTC approach has not realised its key benefits compared to the “integrated” approach with our data. The most striking difference between the maps obtained with both approaches is the presence of a ninth linkage group (linkage group 4) in the map derived from the “integrated” approach. Since markers located on linkage group 4 show a strong cross-link to linkage group 2, it is assumed that both linkage groups are located on the same chromosome. As the grouping in the eight linkage groups and calculated marker orders were mainly stable independent of the mapping approach, calculated maps can be assumed to be close to the true chromosomal arrangement in *C*. *vulgaris*. The major advantage of ML mapping compared to the RG algorithm is its reduced sensitivity to missing data, because neighbouring markers are used for approximation [30]. This is also beneficial when using not fully informative markers for genetic map construction [14] since the data set in this study was complete for all genotypes. In cross-pollinating species, the data set contains markers with different segregation types. Using RG mapping, markers with a different segregation type may not provide their information to their next neighbouring marker, because the next informative neighbour of the same segregation type might not be identical with the closest neighbour [15]. In JoinMap 4.1, ML mapping was clearly slower and computationally more demanding than RG mapping. Furthermore, the addition of distorted markers to the data set was obviously penalised by extreme map distances in ML mapping, because missing data or genotyping errors provoke non-existing recombination which increased map distances [30]. This effect can be used to detect highly error-prone markers, because these will be isolated by large gaps from neighbouring markers [30]. Excellent examples are marker h2m11_121 on linkage group 9 or h6m15_201 on linkage group 8. On linkage group 3, even a smaller cluster on top of the linkage group (Figure 2) ought to be removed accordingly. However, also in RG mapping, poorly fitting markers are expected to stand out [15]. Consequently, the position of the marker h1m4_125 on linkage group 5 from the PTC approach indicates poor fitting (Figure 2). The extreme increase of map length using the ML algorithm due to the addition of distorted markers is another hint of technical deficiencies (e.g. marker complexes) as the reason for the segregation distortion. The ML algorithm maps any marker arrangement whereas RG mapping leaves interfering markers unmapped. Therefore, the reduction of the data in the present study to undistorted markers clearly improved the mapping result of ML mapping.

Apart from map length, map order was also influenced by the chosen mapping algorithm. Inversions in map order in RG maps are mostly caused by changed positions of less informative biparental markers (3:1 segregation), since in the dominant AFLP marker system, it is impossible to distinguish heterozygous (+−) and homozygous (++) loci, making it impossible to assign heterozygous markers to either parent. Thus, these markers cannot provide their full information content which makes their localisation on the map dubious in “integrated” RG mapping [31,32] as well as in the PTC approach [33]. In addition, in mapping populations with about 100 members, inversions in map order caused by the problematic ratio of population size to marker saturation can be an issue, since only a limited number of recombination events can be examined. Consequently, a total number of 200 individuals for all types of mapping populations is recommended to construct reliable linkage maps [34].

## Conclusions

In summary, in the present study on mapping AFLP markers in *C*. *vulgaris*, we prefer the “integrated” mapping approach compared to the PTC approach, since it incorporates more loci which makes estimation of linkage groups more reliable. Using this “integrated” mapping strategy, distorted markers were initially kept in the data set and their use refused after checking their localization on the maps. RG mapping was superior to ML mapping due to the increase of map length using the ML algorithm; benefits of the ML algorithm could not be realised due to the quality of the marker data.

Therefore, the presented “integrated” RG map in Figure 2 is assumed to be the best approximation of the genetic structure of *C*. *vulgaris*. Since the AFLP marker h2m11_157 mapped without any recombinants at the same locus as the trait “flower type”, this marker can be used for marker-assisted selection of this economically most important breeding target in *C*. *vulgaris*. The presented map can also serve as basis for map-based cloning to elucidate the genetic background of the unique flower architecture of bud-blooming *C*. *vulgaris*.

## Methods

### Plant material

The mapping population resulted from a backcross of the cultivar ‘Maria’ x ‘F1’, ‘F1’ being the offspring of a cross of the cultivars ‘Maria’ x ‘Boskoop’. ‘Maria’ is a bud-bloomer with green foliage and white flowers. ‘Boskoop’ has pink flowers with wild-type morphology and yellow foliage. Since the bud-flowering phenotype is inherited recessively, ‘F1’ displays wild-type flower architecture. Due to the lack of stamens, ‘Maria’ served as female crossing partner and was pollinated with freshly-collected pollen of the male crossing partner ‘F1’. The progeny comprised 124 plants. It was segregating 1:1 with regard to the phenotypical traits “flower type”, “flower colour”, and “leaf colour”. Plants were cultivated in pots in the greenhouse during winter and under field conditions in frost-free seasons.

### DNA extraction

Young leaf material was collected from adult plants. 200 mg shock-frozen plant material was ground with a Retsch Tissue Lyser (Qiagen) under continuous cooling. Genomic DNA was extracted using the DNeasy Plant Mini Kit (Qiagen, Hilden) according to the manufacturer’s instructions. DNA was quantified via a Qubit Fluorimeter (Invitrogen).

### AFLP procedure

The AFLP procedure was conducted and its reproducibility tested according to [10]. MseI, HhaI, and HindIII were used to digest diluted DNA. Using two or three selective bases at the 3′-end of each the HindIII, MseI and HhaI primers, the resolution of AFLP gels was most effective. 43 MseI/HindIII and 28 HhaI/HindIII primer combinations were used (Additional file 4).

### Marker scoring

Scoring of polymorphic markers and band size determination was performed with the automated AFLP analysis software SAGA 3.3 (Licor). Bands were recorded as + (present) and – (absent). The markers were named in a trinomial term: code of the HindIII primer (h1-h10), code of the MseI primer (m1-m17), code of the HhaI primer (hh1-hh8) respectively, and corresponding band size in base pairs. An example for the resulting name is “h2m1_142”, a marker generated with the primer pair “HindIII-2” and “MseI-1” with a size of 142 bp. Biparental markers were indicated by the prefix bp (biparental). The stable phenotypical markers “flower colour” (*flowercolour*), “flower type” (*flowertypewt*) and “leaf colour” (*leafgreen*/*leafyellow*) were scored visually during phenotyping in autumn 2007 in six clones per genotype. Phenotyping was repeated in 2011 in six clones per genotype obtained from cuttings. Scoring of the trait “colour of the shoot tip” (*shoottipblushed*) was done only in 2011. Leaf colour was coded either as *leafgreen* or *leafyellow* and served as internal control, because it was assumed that both phenotypes are encoded by alleles of the same gene. Thus, the markers *leafgreen* and *leafyellow* are supposed to be positioned at the same locus. The same assumption was made for *flowercolour* and *shoottipblushed* which are both depending on anthocyanin biosynthesis.

### Segregation of markers

All markers were analysed for their goodness of fit using a *χ*^2^-test (α = 0.05). For maternal and paternal markers, a segregation ratio of 1:1 and for biparental markers a segregation ratio of 3:1 was expected. Markers with other segregation ratios were categorized as odd. Those were initially excluded from the data set and later added in groups: First, maternal and paternal markers with 3:1 and biparental markers with 1:1 segregation were included; secondly, all markers with any segregation ratio were added. From this procedure, three data sets resulted for further analysis: (i) markers with the expected segregation ratios as described above (undistorted segregation), (ii) all markers segregating 1:1 and 3:1, (iii) all markers.

### Mapping approaches

Genetic maps were calculated using the JoinMap 4.1 software [15,33]. Since the mapping population resulted from a cross between two heterogeneously heterozygous and homozygous diploid parents, the “cross-pollination” (CP) mode was used. The data set was either transferred completely (“integrated” approach) or separated into a maternal and a paternal data set for map construction using the “two-way pseudo-testcross” (PTC) approach. For heterozygous cross-pollinating parents, the construction of individual maps according to the PTC mapping approach [35] is often favoured because of plainer linkage phase estimation and clearer attribution of segregation distortion to one parent [32]. For the PTC approach, grouping and linkage phase determination was done independently for the parental data sets followed by map integration with biparental markers serving as anchor markers.

### Estimation of linkage groups and mapping algorithms

Markers with identical segregation patterns were excluded from the data set. Linkage groups were estimated by applying independence LOD threshold ranges from 2 to 15. The initial groups were selected from the groupings tree by choosing nodes with a LOD from 3 to 12. These were checked preliminarily, if a regression map could be established using the standard calculation options of JoinMap 4.1: recombination frequency < 0.45, LOD >1, goodness-of-fit jump 3, ripple after three loci. By examining the strongest cross-link (SCL), related LOD and grouping values, ungrouped markers were manually transferred to groups and the grouping repeated. If mapping was not possible, linkage groups with a higher LOD score were chosen. Markers disturbing the grouping were excluded. In the PTC approach, insufficiently linked markers were tested separately to examine if they were linked to each other [36]. Genetic distances were calculated based on recombination frequencies according to [37]. “Integrated” maps were constructed using regression mapping (RG) or the multipoint maximum likelihood (ML) mapping algorithm [38] modified for full-sib families of outbreeding species [33]. The PTC approach was combined with RG mapping only. JoinMap’s option to force conflicting markers onto the map in a third round of map construction was not used. Only maps from the first round of mapping were considered for further analysis, as results from the second round were not obtained for all map calculations, and because maps from the first and second round (if available) differed only marginally. The calculation of genome coverage was performed according to [39] described in [40].

### Availability of supporting data

All data sets supporting the results of this article are included within the article and its additional files.

## Competing interests

The authors declare that they have no competing interests.

## Authors’ contributions

AB carried out the mapping analysis and drafted the manuscript. MS participated in map construction. AH and TB conceived the study. AH and MS critically revised the manuscript. All authors read and approved this final manuscript version.

## Supplementary Material

Additional file 1 **Main characteristics of linkage groups resulting from the “integrated” mapping approach combined with the RG mapping algorithm.** Table that summarizes size, loci number, and number of distorted markers (in brackets) are given. Groups of markers displaying different segregation ratios have been added stepwise (data set 1: only markers displaying the expected segregation ratios; data set 2: all markers segregating 1:1 and 3:1; data set 3: all markers). Click here for file

Additional file 2 **Main characteristics of linkage groups resulting from the PTC mapping approach combined with the RG mapping algorithm.** Table that summarizes size, loci number, and number of distorted markers (in brackets) are given. Groups of markers displaying different segregation ratios have been added stepwise (data set 1: only markers displaying the expected segregation ratios; data set 2: all markers segregating 1:1 and 3:1; data set 3: all markers). Click here for file

Additional file 3 **Main characteristics of linkage groups from the “integrated” mapping approach combined with the ML mapping algorithm.** Table that summarizes size, loci number, and number of distorted markers (in brackets) are given. Groups of markers displaying different segregation ratios have been added stepwise (data set 1: only markers displaying the expected segregation ratios; data set 2: all markers segregating 1:1 and 3:1; data set 3: all markers). Click here for file

Additional file 4 **Primer combinations used for generation of AFLP markers.** Table with primer combinations and resulting marker codes. Click here for file
