# Supplementary material for: AFLP-based genetic mapping of the “bud-flowering” trait in heather (Calluna vulgaris)
Source: BMC Genet. 2013 Aug 2;14:64. doi: 10.1186/1471-2156-14-64 (PMC3751046; doi:10.1186/1471-2156-14-64)
Supplement: Additional file 3 — Main characteristics of linkage groups from the “integrated” mapping approach combined with the ML mapping algorithm. Table that summarizes size, loci number, and number of distorted markers (in brackets) are given. Groups of markers displaying different segregation ratios have been added stepwise (data set 1: only markers displaying the expected segregation ratios; data set 2: all markers segregating 1:1 and 3:1; data set 3: all markers). [file 1471-2156-14-64-S3.docx]

|  | Data set 1 | | 1 versus 2 | Data set 2 | | 2 versus 3 | Data set 3 | |
| --- | --- | --- | --- | --- | --- | --- | --- | --- |
| Linkage group | Length in cM | Number of loci | Common loci | Length in cM | Number of loci | Common loci | Length in cM | Number of loci |
| 1 | 616 | 56 | 89% | 15544.6 | 58 (7) | 88% | 10788.1 | 71 (17) |
| 2 | 173.7 | 22 | 60% | 276.9 | 41 (18) | 93% | 373.2 | 43 (19) |
| 3 | 10283.8 | 40 | 73% | 10555.7 | 71 (12) | 80% | 20847.4 | 103 (22) |
| 4 | 111.4 | 28 | 92% | 5140.7 | 32 (2) | 72% | 10318 | 27 (8) |
| 5 | 196.1 | 26 | 92% | 252.2 | 26 (1) | 94% | 243.6 | 29 (2) |
| 6 | 464.3 | 40 | 71% | 5733.1 | 73 (13) | 89% | 10692.6 | 91 (25) |
| 7 | 264.5 | 50 | 70% | 15576.3 | 81 (21) | 85.3% | 15772.5 | 110 (19) |
| 8 | 218.2 | 27 | 89% | 510.9 | 36 (8) | 75% | 471.4 | 39 (13) |
| 9 | 242.1 | 28 |  |  |  |  |  |  |
| total | 12570.1 | 321 | 63% | 48334.7 | 418 (90) | 78% | 64299.7 | 510 (125) |
| Genome coverage | 227.7% | |  | 26.9% | |  | 37.9% | |
| cM/Mb ratio | 109.3 | |  | 420.3 | |  | 559.1 | |
