# Supplementary material for: AFLP-based genetic mapping of the “bud-flowering” trait in heather (Calluna vulgaris)
Source: BMC Genet. 2013 Aug 2;14:64. doi: 10.1186/1471-2156-14-64 (PMC3751046; doi:10.1186/1471-2156-14-64)
Supplement: Additional file 4 — Primer combinations used for generation of AFLP markers. Table with primer combinations and resulting marker codes. [file 1471-2156-14-64-S4.pdf]

Table 1: Primer combination used for generation of AFLP markers

MseI/HindIII primer combinations	HhaI/HindIII primer combinations
MseI-AGC-HindIII-CAT	HhaI-AC-HindIII-ACA
MseI-AGC-HindIII_ACA	HhaI-AC-HindIII-CAT
MseI-CAG-HindIII-CAT	HhaI-CA-HindIII-CAT
MseI-CAG-HindIII_ACA	HhaI-CA-HindIII-ACA
MseI-TCT-HindIII-CGA	HhaI-CT-HindIII-CAT
MseI-TCT-HindIII-AGT	HhaI-CT-HindIII-ACA
MseI-TCG-HindIII-CAT	HhaI-CAA-HindIII-ACA
MseI-TCG-HindIII-ACA	HhaI-CAA-HindIII-CAT
MseI-CT_HindIII-CGA	HhaI-CAC-HindIII-CAT
MseI-CT_HindIII-AGT	HhaI-CAC-HindIII-CAT
MseI-CGA-HindIII-AGT	HhaI-CC-HindIII-CAT
MseI-CGA-HindIII-ATC	HhaI-CC-HindIII-ACA
MseI-AGT-HindIII-CGA	HhaI-AA-HindIII-CGA
MseI-AGT-HindIII-AGT	HhaI-AC-HindIII-CGA
MseI-CAC-HindIII-CA	HhaI-CA-HindIII-AGT
MseI-CAC-HindIII-AC	HhaI-CA-HindIII-CGA
MseI-CAC-HindIII-CAT	HhaI-CA-HindIIII-AGT
MseI-CAC-HindIII-ACA	HhaI-CC-HindIII-CGA
MseI-GAT-HindIII-ACA	HhaI-CC-HindIII-AGT
MseI-GAT-HindIII-CAT	HhaI-CT-HindIII-CGA
MseI-TCA-HindIII-CGA	HhaI-CT-HindIII-AGT
MseI-TCA-HindIII-AGT	HhaI-CG-HindIII-CGA
MseI-CAG-HindIII-CA	HhaI-CG-HindIII-AGT
MseI-CAG-HindIII-AC	HhaI-AA-HindIII-AAC
MseI-GTA-HindIII-CAT	HhaI-CA-HindIII-AAC
MseI-GTA-HindIII-ACA	HhaI-CA-HindIII-ACT
MseI-TCC-HindIII-CAT	HhaI-CAA-HindIII-CGA
MseI-TCC-HIindIII-ACA	HhaI-CAA-HindIII-AGT
MseI-CAA-HindIII-CAT	
MseI-TCC-HindIII-CGA	
MseI-TCC-HindIII-AGT	

MseI-TCG-HindIII-CA	
MseI-TCG-HindIII-AC	
MseI-GTG-HindIII-CGA	
MseI-GTG-HindIII-AGT	
MseI-TAC-HindIII-CA	
MseI-TAC-HindIII-AC	
MseI-TAC-HindIII-CGA	
MseI-TAC-HindIII-AGT	
MseI-AT-HindIII-CAT	
MseI-AT-HindIII-ACA	
MseI-GAC-HindIII-CAT	
MseI-GAC-HindIII_ACA	
